# Incidence and outcome of contrast-associated acute kidney injury assessed with Risk, Injury, Failure, Loss, and End-stage kidney disease (RIFLE) criteria in critically ill patients of medical and surgical intensive care units: a retrospective study

**DOI:** 10.1186/s12871-015-0008-x

**Published:** 2015-03-03

**Authors:** Myoung Hwa Kim, Shin Ok Koh, Eun Jung Kim, Jin Sun Cho, Sung-Won Na

**Affiliations:** 1 Department of Anesthesiology and Pain Medicine, Anesthesia and Pain Research Institute, Yonsei University College of Medicine, 50 Yonsei-ro, Seodaemun-gu, Seoul, 120-752 Republic of Korea; 2 Department of Anesthesiology and Pain Medicine, Chung-Ang University College of Medine, Seoul, Republic of Korea

**Keywords:** Contrast-associated acute kidney injury, Intensive care unit, RIFLE classification

## Abstract

**Background:**

Contrast medium used for radiologic tests can decrease renal function. However there have been few studies on contrast-associated acute kidney injury in intensive care unit (ICU) patients. The objective of this study was to evaluate the incidence, characteristics, and outcome of contrast-associated acute kidney injury (CA-AKI) patients using the Risk, Injury, Failure, Loss, and End-stage kidney disease (RIFLE) criteria in critically ill patients in the ICU.

**Methods:**

We conducted a retrospective study of adult patients who underwent contrast-enhanced radiologic tests from January 2011 to December 2012 in a 30-bed medical ICU and a 24-bed surgical ICU.

**Results:**

The study included 335 patients, and the incidence of CA-AKI was 15.5%. The serum creatinine and estimated glomerular filtration rate values in the CA-AKI patients did not recover even at discharge from the hospital compared with the values prior to the contrast use. Among 52 CA-AKI patients, 55.8% (n = 29) had pre-existing kidney injury and 44.2% (n = 23) did not. The CA-AKI patients were divided into risk (31%), injury (31%), and failure (38%) by the RIFLE classification. The percentage of patients in whom AKI progressed to a more severe form (failure, loss, end-stage kidney disease) increased from 38% to 45% during the hospital stay, and the recovery rate of AKI was 17% at the time of hospital discharge. Because the Acute Physiology and Chronic Health Evaluation (APACHE) II score was the only significant variable inducing CA-AKI, higher APACHE II scores were associated with a higher risk of CA-AKI. The ICU and hospital mortality of patients with CA-AKI was significantly higher than in patients without CA-AKI.

**Conclusions:**

CA-AKI is associated with increases in hospital mortality, and can be predicted by the APACHE score.

**Trial registration:**

NCT01807195 on March. 06. 2013.

## Background

Despite advances in supportive care measurements, acute kidney injury (AKI) remains one of the major causes of mortality and morbidity in critically ill patients in intensive care units (ICUs) [1]. There are many factors in ICU patients that can impair renal function, such as septicemia, hypotension, and the use of drugs causing renal dysfunction [2]. In these factors, contrast agents have been shown to be one of the significant causes of AKI; however, their use is unavoidable in the diagnosis and treatment of this patient population, and many studies have investigated this complication. Seelinger et al. [3] reported that mechanisms of renal injury include direct cytotoxic effects, autocrine and paracrine factors that perturb renal hemodynamics, altered rheological properties that affect renal hemodynamic and tubule-dynamics, and regional hypoxia. However, the pathophysiology underlying contrast-associated acute kidney injury (CA-AKI) is still not completely understood.

The incidence of CA-AKI in critically ill patients ranges from less than 2% to 18%, depending on the study populations and the definition of CA-AKI [4-8]. In one study, acute renal failure in critically ill patients was associated with increased use of hospital resources and increased morbidity and mortality due to the limited resources of the ICU [9]. In addition, with CA-AKI, 64% mortality was reported in adult ICU patients requiring renal replacement therapy (RRT) [10].

The RIFLE (Risk, Injury, Failure, Loss, and End-stage kidney disease [ESKD]) criteria are based on elevated serum creatinine (SCr) and decreased estimated glomerular filtration rate (eGFR) and urinary output (UO) from baseline; these criteria have been used to define AKI and classify patients according to the severity of AKI [11]. The RIFLE classification was developed by the Acute Dialysis Quality Initiative group in 2004; it includes the progressive severity classes risk, injury, and failure, as well as two outcome classes: loss and ESKD [12].

The aim of this study was to evaluate the incidence of CA-AKI using the RIFLE criteria, the clinical outcome of renal function, and risk factors for CA-AKI in ICU patients from 2011 to 2012. Additionally, we assessed the relationship of CA-AKI to the outcome of ICU patients, such as the requirement for RRT, the duration of stay and mortality rate in the ICU and hospital.

## Methods

We conducted a retrospective study with patients who received CM for computed tomography (CT) or magnetic resonance imaging (MRI) from January 2011 to December 2012 in a 30-bed medical ICU and a 24-bed surgical ICU at a single university-affiliated hospital. This study was approved by the Institute of Research Committee at Severance Hospital, Yonsei University Health System (IRB number: 4-2012-0922) and registered as a clinical trial (NCT01807195). Informed consent from patients and families was waived due to the retrospective nature of the review of electronic hospital records.

### Data collection

We collected the following variables from patients excluding those under 18 years of age.

- We collected baseline patients’ characteristics: age, gender, body height and weight, Acute Physiology and Chronic Health Evaluation (APACHE) II score, admission categories (medical or surgical), admission diagnosis (cardiovascular, respiratory, neurologic, renal, operation, etc.), and co-morbidities (hypertension, diabetes mellitus, cardiac disease, liver disease, respiratory disease, kidney disease).
- Blood urea nitrogen (BUN), SCr and eGFR values were measured the day of the radiologic study, immediately prior to the administration of CM (baseline), and thereafter at 24 hours, 48 hours, 72 hours, and on the ICU discharge day and hospital discharge day. UO was also measured at the same time periods.
- We investigated variables suspected of being risk factors for CA-AKI based on previous studies: the type of radiologic tests, the volume of CM, mean arterial pressure (MAP) and hemoglobin (Hb) values at the point of contrast use, preventive measures (N-acetylcysteine and isotonic crystalloid) before and after CM administration, whether RRT was applied, and whether diuretics were used after contrast administration.

### Outcomes

The primary outcomes were the incidence of CA-AKI and the progress of CA-AKI using the RIFLE classification (Table 1) [11]. We defined CA-AKI with the RIFLE criteria as a relative increment in SCr of ≥ 50%, or a relative decrement in eGFR of ≤ 25% from baseline, or an episode of oliguria lasting ≥ 6 h within 48 to 72 hours following contrast administration. Secondary outcomes were risk factors for CA-AKI and the requirement for RRT, length of stay in the ICU and hospital after contrast use, and mortality rate in the ICU and hospital.

**Table 1:** **RIFLE (Risk, Injury, Failure, Loss, End-stage kidney disease) criteria for the definition of acute kidney injury**

RIFLE category	GFR criteria	Urine output criteria
Risk	Increased serum creation × 1.5 or decreased of GFR >25%	Urine output <0.5 mL/kg/hr for 6 hrs
Injury	Increased serum creatinine × 2 or decrease of GFR >50%	Urine output <0.5 mL/kg/hr for 12 hrs
Failure	Increased serum creatine × 3 or decrease of GFR >75% or serum creatinine ≥ 4 mg/dL	Urine output <0.3 mL/kg/hr for 12 hrs or anuria for 12 hrs
Loss End-stage kidney disease	Complete loss of renal function for >4 wks Need for RRT for >3 mos.	

GFR, glomerular filtration rate; RRT, renal replacement therapy.

### Statistical analysis

The occurrence of CA-AKI was examined, and patients developing CA-AKI were compared with a group of patients who did not develop AKI after CM use. For continuous variables, all statistical values were expressed as the mean (standard deviation), or median (minimum-maximum). T-test and Mann–Whitney U test were used when comparing groups. For nominal variables or categorical variables, Chi-square test and Fisher’s exact test were used. A binary logistic regression model and the area under the curve (AUC) for the receiver operating characteristic (ROC) curve were used to analyze the various factors that affect renal function decline and to find predictors for the occurrence of CA-AKI. The change in BUN, SCr, eGFR and UO depending on time were assessed with repeated measures ANOVA after Bonferroni’s post hoc test.

The results are shown as adjusted odds ratios with 95% confidence intervals (CI). All *p* values were two-tailed, and *p* value of less than 0.05 was considered statistically significant. Statistical analyses were performed using a statistical software package (SPSS, version 20 for windows, Chicago, IL, USA).

## Results

During the 2-year study period, 4561 patients were admitted to the ICU, and 542 CT and MRI studies (enhanced and non-enhanced) were performed in this population. 359 patients received contrast for radiologic studies, and 24 patients under 18 years of age were excluded. As a result, 335 patients were evaluated for CA-AKI. The CM used in this study was non-ionic and low-osmolality in nature.

1. Demographic data of the patients with and without CA-AKI
  Fifty-two of the 335 patients evaluated (15.5%) developed CA-AKI. In our study, ICU patients were admitted to address the respiratory system problems in CA-AKI and CA-NAKI groups. The APACHE II scores were significantly higher in patients with than without CA-AKI (median [interquartile range]: 17 [13-17] vs. 15.5 [13-16,18,19]; *p* = 0.001, Mann–Whitney U test). However, there were no significant differences between the two groups regarding age, gender, BMI, surgery or not, diagnosis, and underlying diseases such as existing kidney disease. At the time of imaging modality using CM, the type of radiographic tests (CT or MRI), volume of CM used, and hemodynamic variables such as MAP and Hb values were not significantly different between the two groups of patients. There was no difference in preventive measures for CA-AKI, such as hydration with isotonic crystalloid and N-acetylcysteine administration within 12 hours before and after CM use (Table 2).

**Table 2:** **Comparison of patients with and without CA-AKI**

	CA-AKI	CA-NAKI	OR	95% CI	*P*
	(n = 52)	(n = 283)			
**Age (years)**	65.5(57–74)	64(53–75)			0.198
**Gender**			0.9	0.51-1.72	0.877
Male	33(63.5%)	19(36.5%)			
Female	175(61.8%)	108(38.2%)			
**Body height (cm)**	162.5(156.1-168.9)	165(158–172)			0.725
**Body weight (kg)**	62.2(46.9-77.5)	61.5(53.5-69.5)			0.215
**ICU unit**			1.3	0.70-2.40	0.426
Medical	32(61.5%)	191(67.5%)			
Surgical	20(38.5%)	92(32.5%)			
**Main department**	General Surgery	Internal Medicine			0.552
	(n = 19, 36.5%)	(n = 117, 41.3%)			
**Admission diagnosis**	Respiratory failure	Respiratory failure			0.922
	(n = 102, 36.0%)	(n = 20, 38.5%)			
**Hypertension**	26(55.0%)	126(44.5%)	1.3	0.69-2.25	0.545
**Diabetic Mellitus**	13(25.0%)	74(26.1%)	0.9	0.48-1.86	1
**Cardiac disease**	18(34.6%)	75(26.5%)	1.5	0.78-2.76	0.241
**Liver disease**	12(23.1%)	59(20.8%)	1.1	0.56-2.31	0.714
**Respiratory disease**	31(59.6%)	143(50.5%)	1.5	0.79-2.64	0.29
**Kidney disease**	118(41.7%)	29(55.8%)	1.8	0.97-3.20	0.069
**Type of tests**					0.416
CT	41(78.8%)	197(69.6%)			
MRI	11(21.2%)	83(29.3%)			
CT & MRI		3(1.1%)			
**CM volume (ml)**	150(125–175)	100(32.5-167.5)			0.09
**APACHE II**	17(15–19)	15.5(13–18)			0.001
**Mean Arterial Pressure (mmHg)**	90.5(70.5-110.5)	86(76–96)			0.056
**Hemoglobin (g/dl)**	9.7(7.4-12.0)	9.4(8.3-10.5)			0.243
**Hydration**	28(53.8%)	135(47.7%)	1.3	0.71-2.31	0.452
**N-Acetylcystein**	16(30.8%)	74(26.1%)	1.3	0.66-2.40	0.499

Data are presented as N (%), median (interquartile range).

*OR* odds ratio, *CI* confidence interval, *P* probability value.

*CA-AKI* contrast-associated acute kidney injury, *CA-NAKI* contrast-associated no acute kidney injury, *ICU* intensive care unit, *CM* contrast medium, *CT* computed tomography, *MRI* magnetic resonance imaging, *APACHE II* Acute Physiology and Chronic Health Evaluation II.
2. Onset and progress of CA-AKI with RIFLE classification
  Onset of CA-AKI occurred 48 hours after contrast use. At baseline, SCr was 1.17 ± 0.8 mg/dl and GFR was 74.3 ± 34.9 ml/min/1.73 m^2^ in these patients. At 48 hours, the values were 1.62 ± 1.09 and 59.9 ± 36.1 respectively (*p* < 0.001). After 72 hours, the increase in creatinine level and decrease in GFR peaked (SCr: 1.83 ± 1.2, GFR: 52.7 ± 33.9; *p* < 0.001). The values for SCr and GFR were 1.54 ± 1.25 and 63.4 ± 33.6, and 1.42 ± 1.14 and 62.4 ± 29.5 at discharge from the ICU and hospital, respectively. Using the RIFLE classification, CA-AKI occurred in 52 patients. Of these, 29 already had decreased renal function before contrast administration and 23 (6.9%) had CA-AKI that developed within 48 hours after contrast use. The 52 CA-AKI patients were divided into Risk (31%), Injury (31%), and Failure (38%) at 72 hours after contrast use and progressed to a more severe form of injury during their stay in the ICU and hospital. The severe form of injury (Failure, Loss, and ESKD) of AKI classification increased from 38% to 45%, and the recovery rate from AKI was 17% (Table 3) at discharge from hospital.

**Table 3:** **The classification of CA-AKI patients by RIFLE criteria from baseline to hospital discharge day in CA-AKI patients**

CA-AKI (N = 52)	Baseline	Contrast use within 72 h	ICU Discharge	Hospital discharge
**Risk**	11(21%)	16(31%)	6(12%)	11(21%)
**Injury**	4(7%)	16(31%)	10(19%)	9(17%)
**Failure**	14(27%)	16(38%)	23(44%)	20(39%)
**Loss**				3(6%)
**ESKD**				
**Recovery**			13(25%)	9(17%)

Data are presented as N (%).

CA-AKI occurred in 52 patients. 30 patients had acute kidney injury before contrast administration already, by RIFLE criteria. Thus, there were new developed 22 CA-AKI patients within 72 hours after contrast use. Baseline means immediately prior to contrast administration.

ICU discharge: average 6.5 days after contrast use.

Hospital discharge: average 29 days after contrast use.

RIFLE classification.

Risk SCr × 1.5, < 0.5 ml/kg/h × 6 h.

Injury SCr × 2, < 0.5 ml/kg/h × 12 h.

Failure SCr × 3, or SCr ≥ 4 mg/dl with an acute rise > 0.5 mg/dl,< 0.3 ml/kg/h × 24 h or anuria × 12 h.

Loss persistent acute renal failure = complete loss of kidney function > 4 weeks.

ESKD End-stage kidney disease > 3 months.

*CA-AKI* contrast-associated acute kidney injury, *ICU* intensive care unit, *RIFLE* Risk, Injury, Failure, Loss, and End-stage Kidney, *SCr* serum creatinine, *ESKD* end stage kidney disease.
3. Risk factor for CA-AKI
  The APACHE II score was a significant variable in CA-AKI, and the AUC (predicted probability) in the ROC curve was 0.63 (95% CI, 0.54–0.71; p = 0.004, *с* statistic) (Figure 1). The cut-off value of the APACHE II score was less than 17. According to the binary logistic regression test, higher APACHE II scores were associated with a higher occurrence risk of CA-AKI (*p* = 0.015, *Exp* (B) = 2.10; 95% CI, 1.15–3.82).

**Figure 1:**
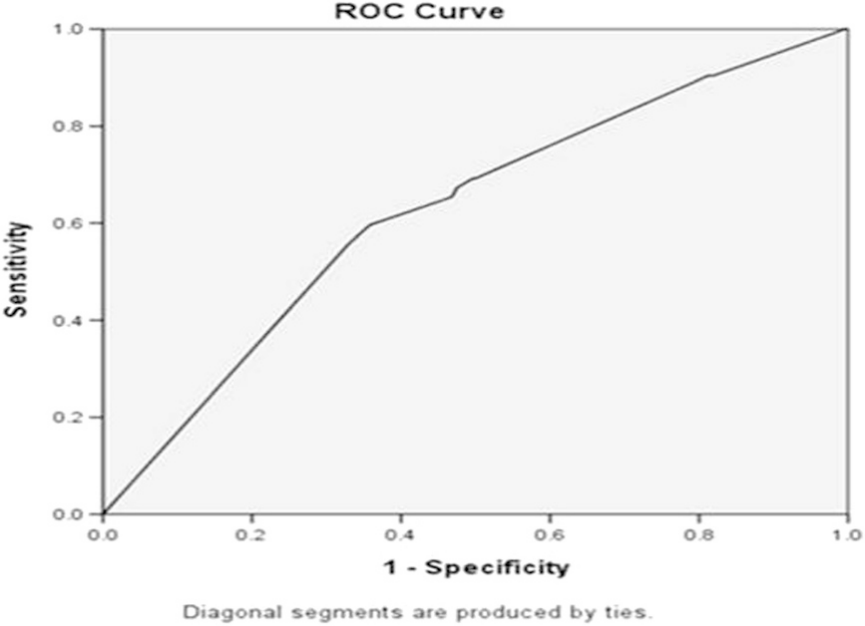
**The ROC curve of the APACHE II score.** AUC (predicted probability of APACHE II score to the CA-AKI) was 0.63 (95% CI: 0.54–0.71, *p =* 0.004, *с* statistic). Cut-off value of APACHE II score was below 17. ROC = receiver operating characteristic, APACHE II *=* Acute Physiology and Chronic Health Evaluation II, AUC = area under ROC curve, CA-AKI = contrast-associated acute kidney injury, CI = confidence interval, *p =* probability value, Cut-off value of APACHE II score > 17.
4. Clinical outcomes after CM administration
  The RRT rate and the frequency of diuretic use in patients with and without CA-AKI were similar. Length of stay (LOS) in the ICU and hospital after contrast administration was also similar between the two groups. The ICU mortality of patients with CA-AKI was significantly higher than in patients without CA-AKI (40.4% vs. 21.3%; OR, 2.51; 95% CI, 1.34–4.67; *p* = 0.005, Chi-square test). Hospital mortality and overall mortality (overall mortality was calculated from the total expired patients including readmission and death thereafter) also were higher in the CA-AKI patients than in patients without CA-AKI (53.8% vs. 35.7%; OR, 2.1; 95% CI, 1.16–3.82; *p* = 0.019/ 55.8% vs. 38.2%; OR, 2.04; 95% CI, 1.12–3.71; *p* = 0.021, Chi-square test) (Table 4). In binary logistic regression analysis, CA-AKI was associated with mortality in the hospital (*p* = 0.015, *Exp* (B) = 2.10; 95% CI, 1.16–3.82). Figure 2 shows the survival of patients with and without CA-AKI during the hospital stay. The survival of CA-AKI patients was significantly lower compared with patients without CA-AKI in the hospital after CM administration (50% survival days in the hospital: 61 days vs. 81 days, Log-Rank test: p = 0.036; Kaplan–Meier plot).

**Table 4:** **Morbidity and mortality in ICU patients after contrast administration**

	CA-AKI	CA-NAKI	OR	95% CI	*P*
	(n = 52)	(n = 283)			
RRT after CM use	18(34.6%)	69(24.4%)	1.6	0.87-3.09	0.13
Diuretics after CM use	37(71.2%)	168(59.4%)			0.12
CM use after admission (days)	4[0–51]	4[0–80]			0.68
ICU discharge after CM use (days)	6.5[1–124]	7[1–285]			0.83
ICU LOS (days)	14[1.5-26.5]	15[6.5-23.5]			0.98
Hospital discharge after CM use (days)	29[1–262]	27[1–651]			0.69
ICU mortality	21(40.4%)	60(21.3%)	2.5	1.34-4.67	0.01
Hospital Mortality	28(53.8%)	101(35.7%)	2.1	1.16-3.82	0.02
Total mortality	29(55.8%)	108(38.2%)	2.0	1.12-3.71	0.02

Data are presented as N (%), median [minimum-maximum].

*OR* odds ratio, *CI* confidence interval, *P* probability value.

*CA-AKI* contrast-associated acute kidney injury, *CA-NAKI* contrast-associated no acute kidney injury, *RRT* renal replacement therapy, *CM* contrast medium, *ICU* intensive care unit, *LOS* length of stay.

**Figure 2:**
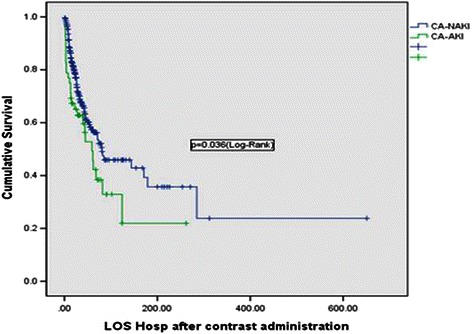
**Kaplan Meier plot.** Kaplan–Meier plot show the survival after contrast medium administration of patients with and without CA-AKI. The survival of CA-AKI patients was significantly lower than the patients without CA-AKI. CA-AKI = contrast-associated acute kidney injury, LOS Hosp = length of stay in hospital after contrast administration.

## Discussion

We evaluated the incidence, characteristics, and outcome of CA-AKI using RIFLE criteria in the ICU population. Because we wanted to determine deterioration of renal function occurring after the use of contrast agents regardless of test types, we investigated AKI after using contrast for either CT or MRI. There have been few studies on CA-AKI using the RIFLE classification in ICU patients, although studies on the incidence of AKI in ICUs with the RIFLE classification have been published [1,12]. Among recent studies, some have been on cardiac ICUs after coronary intervention or cardiopulmonary bypass surgery [13,14]. Additionally, discrepant results have been reported regarding the incidence and outcome of CA-AKI in medical or surgical ICUs [4-8].

In our study, the incidence of CA-AKI was 15.5%, similar to other studies [6,15]. We also found that SCr increased and GFR decreased at 48 hours after contrast use, and peaked at 72 hours. Rashid et al. reported a CA-AKI rate of 11.5%, defined as both the absolute and relative increments of plasma creatinine within 48 to 72 hours after intravenous CM injection for CT [6]. Christophe et al. reported a rate of 16.8%, defining CA-AKI as both the absolute and relative increments of plasma creatinine within 48 hours. Additionally, the onset of CA-AKI varied from 2 days to even 1 week after the procedures, but was usually assessed within 48 hours [15]. We monitored SCr and GFR during patients’ stays in the ICU and hospital, and they had not recovered to pre-contrast levels even at the time of discharge from the ICU or hospital.

Twenty-nine of the CA-AKI patients already had renal dysfunction before contrast use. The recovery of AKI at hospital discharge was only 17%, and this was lower compared with that seen on ICU discharge (25%). In addition, high-severity subgroups based on the RIFLE classification (Failure, Loss, and ESKD) constituted 45% of the CA-AKI patients at the time of hospital discharge. According to the RIFLE classification, patients with CA-AKI progressed toward a more severe decrease in kidney function, and it was found that renal function in CA-AKI was unlikely to recover compared with function prior to contrast use. This progress of CA-AKI was also shown by the SCr and GFR changes. In another report, patients with RIFLE class I or class F incurred a significantly increased length of stay and an increased risk of hospital mortality compared with those who did not progress past class R or those who never developed AKI, even after adjusting for baseline factors such as severity of illness, case mix, race, gender, and age [1]. The RIFLE criteria afford a good opportunity for AKI researchers to compare the incidence of AKI, early diagnosis of AKI, interventional studies to prevent the development or to facilitate the recovery process of AKI, and prediction of AKI outcomes [13,16,17]. Moreover, the RIFLE criteria represent a simple tool for the detection and classification of AKI and for correlation with clinical outcomes [18]. For these reasons, we used the RIFLE classification to describe CA-AKI.

The APACHE II score was the only significant variable in the development of CA-AKI in our study. The APACHE II score is a tool used to determine patients’ severity of critical illness, and this scoring is widely used with the Simplified Acute Physiology Score II in ICUs [2,6]. Although it is not clear whether the APACHE II score is useful in clinical practice as a predictor of CA-AKI, it is certain that the more critically ill the patients in ICUs are, the more vulnerable they may be to AKI; thus, a more active prevention and treatment is needed for CA-AKI. In a recent study [19], the severity of disease and the global nephrotoxic burden were risk factors for AKI, regardless of iodinated CM infusion. The toxic effect of modern CM appeared minimal but because it contributes to the overall nephrotoxic burden, preventive measures should still be considered at the time of CM infusion, at least in high-risk patients. That is, ICU patients have a higher severity of illness than general hospital patients, and are more susceptible to CA-AKI. Further research is needed for ICU populations such as those in this study. Therefore, it is very important to continue using known preventive procedures for CA-AKI, but further research is needed to discover more effective preventive methods. It is also important to improve the overall conditions of ICU patients prior to testing with contrast in order to reduce the severity of illness.

Risk factors for CA-AKI are understood for patients undergoing percutaneous coronary angiography [20], but have not been clarified in medical or surgical ICUs [6]. Our analysis identified risk factors that may assist in predicting patients at risk of developing CA-AKI in the ICU. We compared two groups with and without CA-AKI using conventional risk factors [16]. Preexisting renal impairment, diabetes mellitus, advanced age, heart failure, hypertension, and amount of CM used are important predisposing factors after percutaneous coronary angiography [21]. In our study, the influence of the volume of CM and application of preventive measures, such as hydration and N-acetylcysteine administration, were not associated with the incidence of CA-AKI. The pathophysiology behind CA-AKI is still not completely shown, although it is thought that renal medullary hypoxia is pivotal to the pathophysiology of CA-AKI [22]. Contrast medium in the medulla affects the fragile balance between oxygen delivery and oxygen consumption through several mechanisms, with the main mechanism being reduced blood perfusion [23]. The molar concentration is a major determinant of two important physicochemical properties of CM solutions: osmolality and viscosity. According to some studies, regardless of CM type, the amount of CM a patient receives is a powerful predictor of CA-AKI [24,25]. Hydration prevents CA-AKI by flushing the tubules, and decreasing the CM dose diminishes tubular fluid viscosity and cytotoxic effects [11]. Additionally, with the possible exception of high-dose N-acetylcysteine [26], no treatment has been unequivocally proven efficient in reducing the CA-AKI risk, and in fact endothelin antagonists may have detrimental effects [27,28].

In our study, the rate of RRT and LOS in the ICU and hospital after contrast use did not show any significant difference. Rihal’s group reported that the risk of RRT, ICU and hospital LOS, and mortality were increased in non-ICU patients developing CA-AKI [26]. Additionally, this risk was higher compared with the incidence of RRT in non-ICU patients with CA-AKI in other studies [9,29]. In our study, the mortality—including in the ICU, hospital, and overall—in CA-AKI patients was higher than seen in previous studies evaluating the prognosis of AKI as defined by the RIFLE criteria [2,18]. In addition, the survival rate of LOS in the hospital after contrast use was significantly reduced for patients with AKI. The reasons for the high mortality rate for CA-AKI could be due to two possibilities: the shortage of aggressive prevention and management for CA-AKI, or the fact that the prognosis of patients with renal failure is poor regardless of contrast use [10].

Our results have several limitations for clinical application to other ICU populations. First, our study was a retrospective analysis at a single center. It may fail to identify patients’ selection biases. The study also had a small sample size. Second, we identified the predictor for CA-AKI as APACHE II but it was not clear whether the CA-AKI was caused by contrast administration, the underlying disease, or both, as AKI may indeed be multifactorial in critically ill patients. Third, we did not investigate further long-term outcomes associated with survival rate based on the administrative patients’ data.

Despite these limitations, we have reported the first significant result in ICU patients to identify the onset and progress of CA-AKI using the RIFLE classification after contrast administration during their stay in the ICU and hospital. We believe our study can provide important advice for a prospective study on CA-AKI in ICUs, including the incidence, relevant risk factors, and further predictors for CA-AKI, and outcomes, resulting in more effective prevention methods.

## Conclusions

The incidence of CA-AKI determined using the RIFLE criteria was 15.5%, and the onset of CA-AKI was within 48 hours after CM administration. The level of creatinine peaked at 72 hours after use, and the recovery rate of the CA-AKI patients as assessed by the RIFLE classification was poor. The APACHE II score was associated with CA-AKI, and CA-AKI was associated with higher mortality in the ICU and hospital. Therefore, while it is helpful to apply thorough preventive measures, such as hydration and N-acetylcysteine administration, in high-risk patients before using CM, further studies are needed to investigate CA-AKI epidemiology, prophylactic strategies, and long-term follow-up of outcomes in critically ill patients in the ICU.
